# A Comprehensive Assessment of Replacing Soybean Meal with Candida Utilis-Fermented Corn Steep Liquor in Broiler Diets: Nutritional Evaluation, Growth Performance, Nutrient Digestibility, Intestinal Health and Serum Biochemical

**DOI:** 10.3390/ani16193117

**Published:** 2026-10-04

**Authors:** Li Chen, Jiankun Feng, Jiamin He, Fang Zhao, Jianmin Yuan

**Affiliations:** 1 State Key Laboratory of Animal Nutrition and Feeding, College of Animal Science and Technology, China Agricultural University, No. 2, Yuanmingyuan West Road, Haidian District, Beijing 100193, China; 13407464748@163.com; 2 Ningxia Eppen Biotech Co., Ltd., Yinchuan 750100, China; hnnd_fjk@126.com (J.F.); hjm18161627220@163.com (J.H.); zf601507115@163.com (F.Z.)

**Keywords:** broiler, fermented corn steep liquor, available energy, standardized ileal amino acid digestibility, growth performance

## Abstract

This study aimed to systematically evaluate the potential of *Candida utilis*-fermented corn steep liquor (CU-FCSL) to replace soybean meal (SBM) in broiler diets. A total of 72 twenty-one-day-old Arbor Acres Plus broilers (six replicates of six birds) were fed basal or test diets (20% substitution) to determine metabolizable energy. The same design was used to determine standardized ileal amino acid digestibility (SID), with an additional nitrogen-free diet group. In a 42-day growth trial, 288 one-day-old broilers were assigned to control, 2%, 4%, or 6% CU-FCSL diets (six replicates of 12 birds). The results showed that CU-FCSL contained AME 11.36 MJ/kg DM and AMEn 11.31 MJ/kg DM, including SID values of Lys (90.00%), Met (94.01%), Thr (84.63%), and Val (88.86%). Compared with the control, broilers fed 2% or 4% CU-FCSL consistently exhibited greater body weight and weight gain across all phases, whereas 6% inclusion impaired growth performance (*p* < 0.05). The 4% level improved ether extract digestibility, immune parameters, and intestinal villus height-to-crypt depth ratio, whereas 6% inclusion increased abdominal fat, elevated serum uric acid and ALT, decreased serum IgG, IgM, and IL-10, and inhibited intestinal development and health. In conclusion, 2% and 4% CU-FCSL could be added to replace SBM without adverse effects on broiler health.

## Simple Summary

Fluctuations in soybean production, global trade uncertainty, and rising feed demand have stimulated the search for alternative protein sources. Corn steep liquor is a cheap by-product from corn processing, characterized by abundant soluble nitrogen compounds, minerals, vitamins, and organic acids. The present study aimed to systematically evaluate whether *Candida utilis*-fermented corn steep liquor safely qualified as a partial substitute for soybean meal in broiler diets. Three experiments were conducted, beginning with the determination of its energy and standardized ileal amino acid digestibility, followed by a 42-day broiler feeding trial to investigate the effects of graded *Candida utilis*-fermented corn steep liquor replacing soybean meal on growth performance, nutrient digestibility, slaughter performance, intestinal health, serum biochemistry, and immune parameters in broilers. Results showed that dietary inclusion of 2% and 4% of this fermented product improved body weight gain and intestinal health. However, a 6% replacement negatively affected weight gain and feed efficiency, increased body fat, and caused signs of intestinal stress. These findings suggested that fermented corn steep liquor with *Candida utilis* is a promising protein ingredient and could partially replace soybean meal at 2% and 4% in broiler diets, improving the use of agricultural waste.

## 1. Introduction

The rapid expansion of intensive poultry production has increased demand for high-quality protein ingredients, while the imbalance between supply and demand for soybean meal (SBM) due to international trade and the pandemic has become a growing concern, stimulating interest in developing sustainable alternatives to conventional protein sources [1,2,3]. Meanwhile, the massive global corn-processing industry produces substantial quantities of nutrient-rich coproducts, which are worth utilizing efficiently as a feed resource to reduce reliance on soybean meal and promote resource recycling [4,5,6]. Among these coproducts, corn steep liquor (CSL) is generated during wet milling and is characterized by abundant soluble nutrients, including soluble proteins, peptides, free amino acids, organic acids, minerals, and growth-promoting compounds [7,8].

Although CSL is widely used as a fermentation substrate [9,10], research on its application in animal feed has increased [11]. The use of conventional CSL in broiler diets has been limited by its inclusion level and product characteristics. Moderate CSL supplementation was generally tolerated and provided nutritional and functional benefits, enhanced growth performance, improved nutrient digestibility, lower gastrointestinal pH, and greater immune responses and antioxidant capacity in broilers, whereas excessive inclusion frequently resulted in adverse effects [12,13,14,15,16,17]. Previous studies showed that increasing CSL supplementation above 10% may result in declines in production performance, suppression of immune organ development [14], histological changes in the gastrointestinal tract with hypertrophy and hyperplasia of the mucosal lining [15], negative nutrient utilization, and adverse carcass characteristics [16]. Then, recent investigations have focused on transforming CSL into more valuable protein ingredients through effective processing technologies to minimize the constraints of conventional CSL. Ebrahimpour et al. evaluated a mixture of probiotics and 4% and 6% dried CSL, which improved humoral immune response and intestinal microbial populations, highlighting the potential contribution of fermentation-derived components in CSL [17]. Guo et al. developed a water-soluble corn protein derived from CSL through integrated processing and demonstrated its potential as a soybean meal substitute at 5% in broiler diets [18]. Similarly, Hajati et al. reported that hydrolytic treatment of CSL could degrade anti-nutritional factors, reduce macromolecular complexity, and enhance intestinal availability of amino acids, peptides, and phosphorus [19]. Microbial fermentation also provided a practical strategy by converting soluble corn-derived nutrients into yeast biomass and fermentation metabolites [20]. However, whether microbial fermentation could increase CSL availability and make it suitable for production remains unclear.

In the present study, *Candida utilis*-fermented corn steep liquor (CU-FCSL), manufactured by liquid-state fermentation, yielded a yellowish-brown powder and represented a biologically upgraded CSL product with potential nutritional advantages. Compared with soybean meal, which required extensive agricultural land and global trade networks, locally available corn-processing coproducts may represent a more efficient protein resource after microbial fermentation. Although the economic competitiveness of CU-FCSL depended on fermentation efficiency, processing costs, and regional availability, its conversion from a low-value agricultural coproduct into a suitable feed ingredient provided a potential strategy for the sustainability of feed resources. Nevertheless, its systematic nutritional evaluation and application in broilers remain to be fully elucidated.

Therefore, the present study hypothesized that CU-FCSL may exhibit enhanced nutritional value and a significant dose-dependent effect. The objective was to systematically evaluate the efficacy of CU-FCSL as a potential alternative protein for broiler diets, determining its apparent metabolic energy (AME) and standard ileal amino acid digestibility (SID). A feeding trial was designed to investigate the effects of graded CU-FCSL replacement of SBM on growth performance, nutrient digestibility, slaughter performance, intestinal health, serum biochemistry, and immune parameters in broilers. The findings were expected to guide the utilization of the corn-processing coproduct CU-FCSL and contribute to the development of sustainable feed resources for poultry production.

## 2. Materials and Methods

All animal procedures were conducted in accordance with the guidelines for the Animal Welfare and Ethics Committee of the Animal Science and Technology College of China Agricultural University (Approval No.:AW71706202-1-103).

### 2.1. Experimental Materials

The test ingredient was provided by Ningxia Eppen Biotech Co., Ltd. (Yinchuan, China) and manufactured by liquid-state fermentation of corn steep liquor using *Candida utilis*, which was named *Candida utilis*-fermented corn steep liquor (CU-FCSL). After fermentation, the whole broth was concentrated and spray-dried without washing or separation. This process resulted in a yellowish-brown powder, whose chemical composition and amino acid profile are presented in Table 1.

### 2.2. Animal Trials

#### 2.2.1. Trial 1: AME Value of CU-FCSL

The substitution (difference) method was used to determine metabolizable energy and amino acid digestibility. In Experiment 1 (Exp. 1), a total of 72 healthy male 21-day-old Arbor Acres Plus (AA+) broilers of uniform body weight were randomly allocated to 2 treatment groups using a single-factor completely randomized design, with 6 replicates per group and 6 birds per replicate. The treatments consisted of a basal diet group and a test diet group in which 20% of the whole basal diet was replaced with CU-FCSL using the substitution method. The basal diet was a corn–soybean meal type diet formulated to meet the nutritional requirements of late-phase broilers by Aviagen [21], whose composition and calculated nutrient levels are presented in Table 2. In the experimental diet, CU-FCSL replaced 20% of the whole basal diet. The experimental period was 7 days, with 4 days of adaptation, followed by 3 days of total fecal collection.

Birds were housed in metabolism cages. After the 3-day adaptation period, birds fasted for 8 h. Ad libitum feeding was then resumed for 64 h, during which feces were collected daily. Feathers and debris were removed, and a 10% hydrochloric acid solution was sprayed to fix nitrogen. Fecal samples were oven-dried at 65 °C to a constant weight. Total feed intake (FI) and total fecal excretion (FE) were recorded. After a 24 h equilibration period, samples were ground using a universal grinder (DE-500g, Hongjingtian Instrument Co., Ltd., Jinhua, China), passed through a 0.425 mm sieve, and stored in sealed bags for gross energy (GE) analysis. The formula for calculating the energy in Exp. 1 is shown below:

$${\mathrm{AME}}_{\mathrm{diet}}\;\left(\mathrm{MJ}/\mathrm{kg},\mathrm{DM}\right)=\;\frac{{\mathrm{GE}}_{\mathrm{diet}}\;\times \mathrm{FI}-{\mathrm{GE}}_{\mathrm{excreta}}\times \;\mathrm{FE}}{\mathrm{FI}}$$

Nitrogen Retention (TRN, g) = CP_diet_ × FI − CP_excreta_ × FE

$${\mathrm{AMEn}}_{\mathrm{diet}}\left(\mathrm{MJ}/\mathrm{kg},\mathrm{DM}\right)=\mathrm{AME}-\frac{\mathrm{TRN}\times 34.39}{\mathrm{FI}}$$

$${\mathrm{AME}}_{\mathrm{ingredient}}(\mathrm{MJ}/\mathrm{kg},\mathrm{DM})={\mathrm{AME}}_{\mathrm{basal}}-\frac{{\mathrm{AME}}_{\mathrm{basal}}-{\mathrm{AME}}_{\mathrm{test}}}{X\%}$$

where GE is the gross energy (MJ/kg, DM), CP is the crude protein content (%, DM), FI is the feed intake (kg, DM), FE is the fecal excretion (kg, DM), and *X*% is the substitution ratio of the test ingredient replacing the basal diet.

#### 2.2.2. Trial 2: SIDAA Value of CU-FCSL

The grouping design for Exp. 2 was the same as Exp. 1, but an extra group with a nitrogen-free diet (6 replicates, 6 birds each) was included to estimate basal ileal endogenous amino acid losses (IEAAL), and detailed ingredients were shown in Table 3.

Each treatment received its respective diet and was fed for 3 days. On day 25, after a 3 h fasting period, a 1 h refeeding followed by a 4 h fasting period, birds were euthanized by electrocution and exsanguination. Terminal ileal digesta, excluding the distal 2 cm adjacent to the ileocecal junction, were collected and pooled per replicate, then freeze-dried using a vacuum freeze dryer (FD-2, Biocool Co., Ltd., Beijing, China) and ground through a 40-mesh sieve. Diet and digesta samples were stored at −20 °C for subsequent analysis of TiO_2_ and amino acids.

The formula for calculating the ileal digestibility of amino acids was shown below:

$${\mathrm{AID}}_{\mathrm{diet}}\left(\%\right)=\left[1-\left(\frac{{\mathrm{AA}}_{\mathrm{digesta}}\times {\mathrm{Ti}}_{\mathrm{diet}}}{{\mathrm{AA}}_{\mathrm{diet}}\times {\mathrm{Ti}}_{\mathrm{digesta}}}\right)\right]\times 100$$

$$\mathrm{IEAAL}\;(\mathrm{mg}/\mathrm{kg}\;\mathrm{DMI})=\frac{{\mathrm{AA}}_{\mathrm{digesta}}\times {\mathrm{Ti}}_{\mathrm{NFD}}}{{\mathrm{Ti}}_{\mathrm{digesta}}}$$

$${\mathrm{SID}}_{\mathrm{diet}}\left(\%\right)={\mathrm{AID}}_{\mathrm{diet}}+\frac{\mathrm{IEAAL}}{{\mathrm{AA}}_{\mathrm{diet}}}$$

$${\mathrm{SID}}_{\mathrm{ingredient}}\left(\%\right)=\frac{{\mathrm{SID}}_{\mathrm{test}}\times {\mathrm{AA}}_{\mathrm{test}}-\left(1-X\%\right)\times {\mathrm{SID}}_{\mathrm{basal}}\times {\mathrm{AA}}_{\mathrm{basal}}}{{\mathrm{AA}}_{\mathrm{test}}-\left(1-X\%\right)\times {\mathrm{AA}}_{\mathrm{basal}}}\times 100$$

where AA was the amino acid concentration (mg/kg DM), Ti was the TiO_2_ concentration (g/kg), IEAAL was the ileal endogenous amino acid loss of the specific amino acid (mg/kg DMI), and X was the substitution ratio of the test ingredient replacing the basal diet (%).

#### 2.2.3. The Effect of Replacing Soybean Meal in Broilers (Trial 3)

##### Experiment Design

A total of 288 1-day-old mixed-sex AA+ broilers of similar initial body weight were randomly allocated to 4 treatment groups. Each treatment consisted of six replicate pens with 12 birds per pen, and an equal proportion of males and females (1:1) was maintained across all pens. The corn–soybean meal basal diet was formulated to meet the nutrient requirements of broilers by Aviagen (Huntsville, AL, USA) for both starter (1–21 d) and grower (22–42 d) phases [21]. Nutrient composition (including AME, CP, Ga, P, AA et al.) of corn, soybean meal, corn gluten meal, and wheat flour was determined by near-infrared scanning prior to formulation. The AME and SID values for CU-FCSL were derived from Trial 1 and Trial 2, respectively. CU-FCSL was included at 2% (CU-FCSL2), 4% (CU-FCSL4), and 6% (CU-FCSL6) of the experimental diet, with isonitrogenous and isocaloric relative to the control. Diet compositions for both phases were presented in Table 4 and Table 5. Titanium dioxide (TiO_2_, 0.5%) was added as an exogenous marker in all diets for digestibility determination. All diets were mixed, pelleted, and crumbled for 1–21 d of age. Representative samples were ground to pass a 0.425 mm sieve and stored sealed for subsequent chemical analysis.

The rearing experiment was carried out at the Poultry Experimental Base of China Agricultural University in Zhuozhou (Hebei, China). All birds were reared according to the AA+ broiler husbandry and management guide, with temperature and lighting maintained at optimal levels and ad libitum access to feed and water throughout the experiment.

On 21 d and 42 d of Exp. 3, one bird from each replicate was selected based on its proximity to the average body weight, while maintaining a balanced sex distribution among treatments. The blood samples were collected from the wing vein and centrifuged at 4 °C, 1500× *g* for 10 min to separate the serum, which was stored at −80 °C until further analyses. Later, these birds were euthanized via electrocution and exsanguination. The thymus, spleen, liver, and bursa of Fabricius were excised and weighed for immune organ index calculation. The jejunum was dissected, and the mucosa was scraped using sterilized microscope slides, snap-frozen in liquid nitrogen, and stored at −80 °C for mRNA expression analysis. A 1 cm piece of mid-jejunal segment was flushed with ice-cold phosphate-buffered saline (PBS) and fixed in 4% paraformaldehyde (Biosharp Life Sciences, Hefei, China) for histomorphology examination. The breast muscle was trimmed along the direction of muscle fibers using a reference scale to obtain uniform strips approximately 30 g to measure drip loss.

On 42 d, another bird per replicate with body weight approximating the mean body weight was chosen and euthanized via electrocution. After feather removal by depilator, slaughter performance was evaluated in accordance with the procedures described in NY/T 823-2020.

##### Determination of Growth Performance

Birds were weighed by replicate after a 12 h feed withdrawal at 21 d and 42 d, and feed weight per pen was recorded. Mortalities and culled birds were recorded daily throughout the experiment, with dead weight incorporated to adjust growth performance parameters. The parameters of feed intake (FI), body weight gain (BWG), and feed conversion ratio (FCR) were calculated as follows:

$$\mathrm{BWG}\;(\mathrm{kg}/\mathrm{bird})=\frac{{\mathrm{BW}}_{\mathrm{final},\;\mathrm{total}}}{{\mathrm{Birds}}_{\mathrm{final}}}-\frac{{\mathrm{BW}}_{\mathrm{initial},\;\mathrm{total}}}{{\mathrm{Birds}}_{\mathrm{initial}}}$$

$$\mathrm{FCR}=\frac{{\mathrm{Feed}}_{\mathrm{initial}}-{\mathrm{Feed}}_{\mathrm{final}}}{{\mathrm{BW}}_{\mathrm{final},\mathrm{total}}+{\mathrm{BW}}_{\mathrm{dead},\mathrm{total}}-{\mathrm{BW}}_{\mathrm{initial},\mathrm{total}}}$$

FI_dead, total_(kg) = BW_dead, total_ × FCR

$$\mathrm{FI}\;(\mathrm{kg}/\mathrm{bird})=\frac{{\mathrm{Feed}}_{\mathrm{initial}}-{\mathrm{Feed}}_{\mathrm{final}}-{\mathrm{FI}}_{\mathrm{dead},\;\mathrm{total}\;}}{{\mathrm{Birds}}_{\mathrm{final}}}$$

##### Drip Loss Measurement

The initial weight (W_1_) of the breast muscle strip was recorded, and each sample was suspended using a fine iron hook at 4 °C for 24 h. After the storage period, surface moisture was gently blotted with qualitative filter paper, and the sample was finally weighed (W_2_). The drip loss was calculated as:

$$\mathrm{Drip Loss}\left(\%\right)=\frac{\mathrm{initial weight}-\mathrm{final weight}}{\mathrm{initial weight}}\times 100$$

##### Apparent Nutrient Digestibility

The exogenous indicator method was applied to measure the digestibility. On days 18 and 39, representative fecal samples were collected per replicate, with 10% hydrochloric acid solution spraying. They were then dried at 65 °C, equilibrated for 48 h, and ground to pass a 40-mesh sieve. Apparent digestibility of crude protein, crude fat, and organic matter was determined by the dietary TiO_2_ indicator dilution method. Nutrient digestibility was determined as:

$$\mathrm{Nutrient Digestibility}\left(\%\right)=\left[1-\left(\frac{{\mathrm{Nutrient}}_{\mathrm{excreta}}\times {\mathrm{TiO}}_{2,\mathrm{diet}}}{{\mathrm{Nutrient}}_{\mathrm{diet}}\times {\mathrm{TiO}}_{2,\mathrm{excreta}}}\right)\right]\times 100$$

##### Chemical Analysis

Conventional nutrients were determined following AOAC (2016) methods. Moisture was determined using a hot-air oven at 105 °C for 5 h (Method 930.15). The EE was determined by Soxhlet extraction (Method 920.39). Ash was determined by Muffle furnace incineration (Method 942.05). The CP analysis was conducted on the KT200 Kjeldahl apparatus (FOSS Analytical A/S, Hillerod, Denmark) as specified by Kjeldahl nitrogen analysis (Method 984.13). Calcium was analyzed by wet ashing–titration (AOAC Method 935.13). Total phosphorus was determined using the molybdenum blue colorimetric method (Method 986.11), and phytate phosphorus was measured following AOAC 986.11 after acid extraction and anion-exchange separation. The amino acid profile was analyzed by high-performance liquid chromatography using AOAC Method 994.12. Total acid was measured by acid–base titration (AOAC 962.16). The 6400 automatic isoperibol calorimeter (Parr Instrument Co., Moline, IL, USA) was used to analyze gross energy via oxygen bomb combustion, in accordance with ISO 9831 (2004). Dietary and excretal TiO_2_ were determined by following established procedures of Myers et al. [22]. Acid-soluble protein was determined according to the method of Cai et al. [23]. Mannan content was determined by high-performance liquid chromatography after acid hydrolysis, following the procedure described by Chen et al. [24]. Fumonisins (B_1_, B_2_, B_3_), zearalenone and T-2 toxin were quantified by LC–MS/MS after immunoaffinity column cleanup, according to AOAC Method 112401 [25].

##### Morphology Analysis

Jejunum tissue slices of 5 μm thickness were obtained after fixing samples using 4% paraformaldehyde and paraffin embedding, followed by hematoxylin–eosin (H&E) staining. Under a Leica DM750 microscope (Leica Microsystems GmbH, Wetzlar, Germany), neatly arranged villus–crypt structures were screened to measure villus height (VH) and crypt depth (CD). VH was defined as the length from the villus–crypt junction to the villus apex, and CD was the distance from the crypt base to the junction. Ten well-oriented villi and crypts per section were measured. Villus height-to-crypt depth ratio (VCR) was calculated as:

$$\mathrm{VCR}=\frac{\mathrm{VH}}{\mathrm{CD}}$$

##### Determination of Serum Biochemistry and Immune Parameters

Serum uric acid (UA), blood urea nitrogen (BUN), aspartate aminotransferase (AST) and alanine aminotransferase (ALT) were quantified by an automatic biochemical analyzer (Chemray 800, Rayto Life and Analytical Sciences Co., Ltd., Shenzhen, China). Immunoglobulin G (IgG), IgA, IgM, interleukin-6 (IL-6), interleukin-10 (IL-10), and C-reactive protein (CRP) were quantified by enzyme-linked immunosorbent assay (ELISA) using commercial kits purchased from Solarbio Science & Technology Co., Ltd. (Beijing, China), according to the manufacturer’s instructions. A microplate reader (Synergy 4, Bio-Tek Instruments Inc., Winooski, VT, USA) was employed to record the optical density (OD) values of all samples.

##### Intestinal mRNA Expression Analysis

Total RNA was extracted from jejunal tissues using TRIzol reagent (Cat. No. 9109, Takara Bio Inc., Kusatsu, Shiga, Japan), and RNA concentration was measured using a NanoDrop 2000 spectrophotometer (Thermo Fisher Scientific Inc., Waltham, MA, USA). The PrimeScript RT reagent kit (RR047A, Takara Bio Inc., Kusatsu, Shiga, Japan) was used for reverse transcription to synthesize complementary DNA (cDNA). Subsequent real-time quantitative PCR (qRT-PCR) was performed with the TB Green Premix Ex Taq kit (RR420A, Takara Bio Inc., Kusatsu, Shiga, Japan) on an Applied Biosystems 7500 Fast Real-Time PCR System (Thermo Fisher Scientific Inc., Foster City, CA, USA). The β-actin gene was selected as the internal reference to normalize relative mRNA expression levels of target genes, adopting the 2^−ΔΔCt^ method. All primer sequences were summarized in Table 6.

### 2.3. Statistical Analysis

All data were analyzed using SPSS 26.0, and graphs were visualized with GraphPad Prism 10. Outliers were identified and excluded using Grubbs’ test, and a total of 4 outliers were excluded across all variables. For comparisons between two groups, an independent-samples t-test was applied. For comparisons among multiple treatment groups, one-way analysis of variance (ANOVA) was performed, followed by Duncan’s multiple comparison test when a significant interaction was detected. Differences were considered statistically significant at *p* < 0.05.

## 3. Results

### 3.1. The Energy Values and Energy Utilization of Diets and CU-FCSL

The effects of dietary CU-FCSL inclusion on energy utilization and nitrogen balance were presented in Table 7. Birds fed the 20% CU-FCSL diet exhibited significantly greater nitrogen excretion compared with the control group (*p* < 0.05), whereas nitrogen intake and nitrogen retention remained unaffected (*p* > 0.05). Dietary AME, AMEn values, and energy utilization of AME were significantly lower in the 20% CU-FCSL diet compared to the control (*p* < 0.05). Based on the substitution method, the calculated AME and AMEn values of CU-FCSL material were 11.36 and 11.31 MJ/kg DM, respectively.

### 3.2. Apparent and Standardized Ileal Digestibility of Amino Acids in CU-FCSL

The apparent ileal digestibility (AID) and standardized ileal digestibility (SID) of amino acids of CU-FCSL were summarized in Table 8 and were determined by the substitution method. Endogenous amino acid losses ranged from 164.81 mg/kg DM for tryptophan to 2141.27 mg/kg DM for glutamic acid. Among the essential amino acids, Met exhibited the highest AID (91.82%) and SID (94.01%), followed by AID_Arg_ (89.21%) and SID_Arg_ (92.65%). Lysine, the most critical limiting amino acid in broiler diets, showed AID and SID values of 85.15% and 90.00%, respectively. Threonine and tryptophan presented relatively lower digestibility among essential amino acids, with SID values of 84.63% and 82.18%, respectively. Notably, this was true among all amino acids examined. For the majority of non-essential amino acids, SID values exceeded 84%, with the exception of cysteine (72.37%) and glycine (80.92%).

### 3.3. Growth Performance

The effects of CU-FCSL on growth performance were summarized in Table 9. During the starter phase (1–21 d), the CU-FCSL6 group showed a trend toward lower final body weight (FBW) and BWG, while the CU-FCSL2 group had the highest numerical values (*p* = 0.072). FCR was significantly affected by dietary treatments with different CU-FCSL levels (*p* < 0.05), with the CU-FCSL2 group showing the most favorable FCR (1.24), which was significantly better than both the control and the CU-FCSL6 group. During this period, FI remained comparable across all groups, indicating that the observed differences in growth performance were primarily attributable to feed efficiency rather than feed consumption. Compared with the control group, the 2% and 4% groups consistently showed greater FBW, and the addition of CU-FCSL significantly increased the BWG of broilers during the 21–42 d phase (*p* < 0.05). A similar pattern was observed over the entire experimental period (1–42 d), with significantly higher values for BWG and FI in the groups supplemented with 2% and 4% CU-FCSL than in the control group, and broilers receiving 2% CU-FCSL consistently achieved the greatest FCR, performing comparably to the control (*p* < 0.05). However, increasing the inclusion level to 6% markedly impaired BWG and FCR, particularly during the starter phase.

### 3.4. Slaughter Traits and Meat Quality

Carcass traits and meat quality of 42 d broilers fed diets containing graded levels of CU-FCSL were presented in Table 10. The dietary treatments did not significantly influence the carcass rate, half-eviscerated rate, eviscerated rate, breast muscle rate and thigh rate (*p* > 0.05). In contrast, dietary treatments also significantly affected abdominal fat percentage and drip loss (*p* < 0.05). The 4% CU-FCSL group showed the lowest abdominal fat percentage 1.42%, comparable to the control, whereas the 2% and 6% groups showed higher values. Regarding meat water-holding capacity, drip loss increased with increasing CU-FCSL inclusion, with the 6% group showing significantly higher drip loss (3.31%) than the control group (*p* < 0.05).

### 3.5. Immune Organ Indexes

The effects of dietary CU-FCSL on immune organ indices in broilers were presented in Table 11. At 21 d of age, spleen index was higher in the CU-FCSL4 group compared with the other treatments (*p* < 0.05). At 42 d of age, thymus index was elevated in the CU-FCSL4 and CU-FCSL6 groups relative to the control and CU-FCSL2 groups (*p* < 0.05). No other significant differences were observed among treatments for liver, bursa of Fabricius, or the remaining immune organ indices.

### 3.6. Intestinal Morphology

The effects of diets involving different levels of CU-FCSL on intestinal morphology parameters at 21 and 42 days of age were shown in Table 12. CD and VCR differed significantly among treatments at 21 d of age (*p* < 0.05). At 42 d of age, crypt depth and VCR remained significantly different among treatments (*p* < 0.05), whereas villus height did not differ (*p* > 0.05). Birds fed the 4% CU-FCSL diet exhibited the greatest intestinal morphology among all groups, whereas the 6% group showed poor performance, which was even worse in comparison to the control group.

### 3.7. Nutrient Digestibility

As demonstrated in Table 13, the digestibility of crude protein (CP), organic matter (OM), and ether extract (EE) was all significantly influenced by dietary CU-FCSL inclusion at 21 d of age (*p* < 0.05). Birds fed the 4% and 6% diets achieved comparable or higher values than the control group, whereas the 2% group exhibited the lowest value. At 42 d of age, CP digestibility was highest in the CU-FCSL6 group, which was significantly greater than other groups (*p* < 0.05). However, no significant differences were detected among treatments for OM or EE digestibility at 42 d of age.

### 3.8. Serum Biochemistry and Immune Parameters

No significant differences were observed among treatments for AST activity or BUN concentration at 21 d of age, but serum ALT activity and uric acid concentration were significantly influenced by dietary treatments (*p* < 0.05, Table 14). ALT activity was elevated in the CU-FCSL2 and CU-FCSL6 groups compared with the control, whereas the CU-FCSL4 group maintained comparable levels to the control. Serum UA concentration at 21 d was numerically higher across all CU-FCSL-supplemented groups relative to the control, with the increase reaching statistical significance in the 2% and 6% groups. The CU-FCSL4 group showed UA levels comparable to the control, with no significant differences. At 42 d of age, BUN and uric acid concentrations were significantly influenced by CU-FCSL supplementation (*p* < 0.05), while ALT and AST activities showed no significant differences among groups (*p* > 0.05). Notably, birds fed the 4% diet displayed the lowest BUN and uric acid concentrations, whereas the 6% group showed the highest uric acid level, consistent with the pattern observed at 21 d of age.

### 3.9. Serum Immune Parameters

The effects of diets involving CU-FCSL on serum immune parameters at 21 and 42 days of age are shown in Table 15 and Table 16.

At 21 d, serum IgG concentration was higher in the CU-FCSL4 group compared with the CU-FCSL6 group (*p* < 0.05), with the control and CU-FCSL2 groups showing intermediate values. Serum IgM concentration was elevated in the CU-FCSL2 group relative to the CU-FCSL4 and CU-FCSL6 groups (*p* < 0.05). Serum IL-10 concentration was elevated in the CU-FCSL4 group relative to the control, CU-FCSL2, and CU-FCSL6 groups (*p* < 0.05). Serum CRP concentration was lower in the CU-FCSL2 group compared with the control and CU-FCSL4 groups (*p* < 0.05), with the CU-FCSL6 group showing intermediate values. No significant difference was observed for IgA and IL-6 (*p* > 0.05).

At 42 d, serum IgG concentration was higher in the CU-FCSL2 group compared with the control and the CU-FCSL4 and CU-FCSL6 groups (*p* < 0.05). Serum IgA concentration was elevated in the CU-FCSL6 group and CU-FCSL2 group relative to the control and CU-FCSL4 groups (*p* < 0.05). Serum IgM concentration was increased in the CU-FCSL2 and CU-FCSL6 groups compared with the control and CU-FCSL4 groups (*p* < 0.01). Serum IL-6 concentration was higher in the CU-FCSL2 and CU-FCSL6 groups relative to the control (*p* < 0.05), with the CU-FCSL4 group showing intermediate values. No significant differences were observed for IL-10 or CRP (*p* > 0.05).

### 3.10. Intestinal Barrier and Immune Function

**Figure 1:**
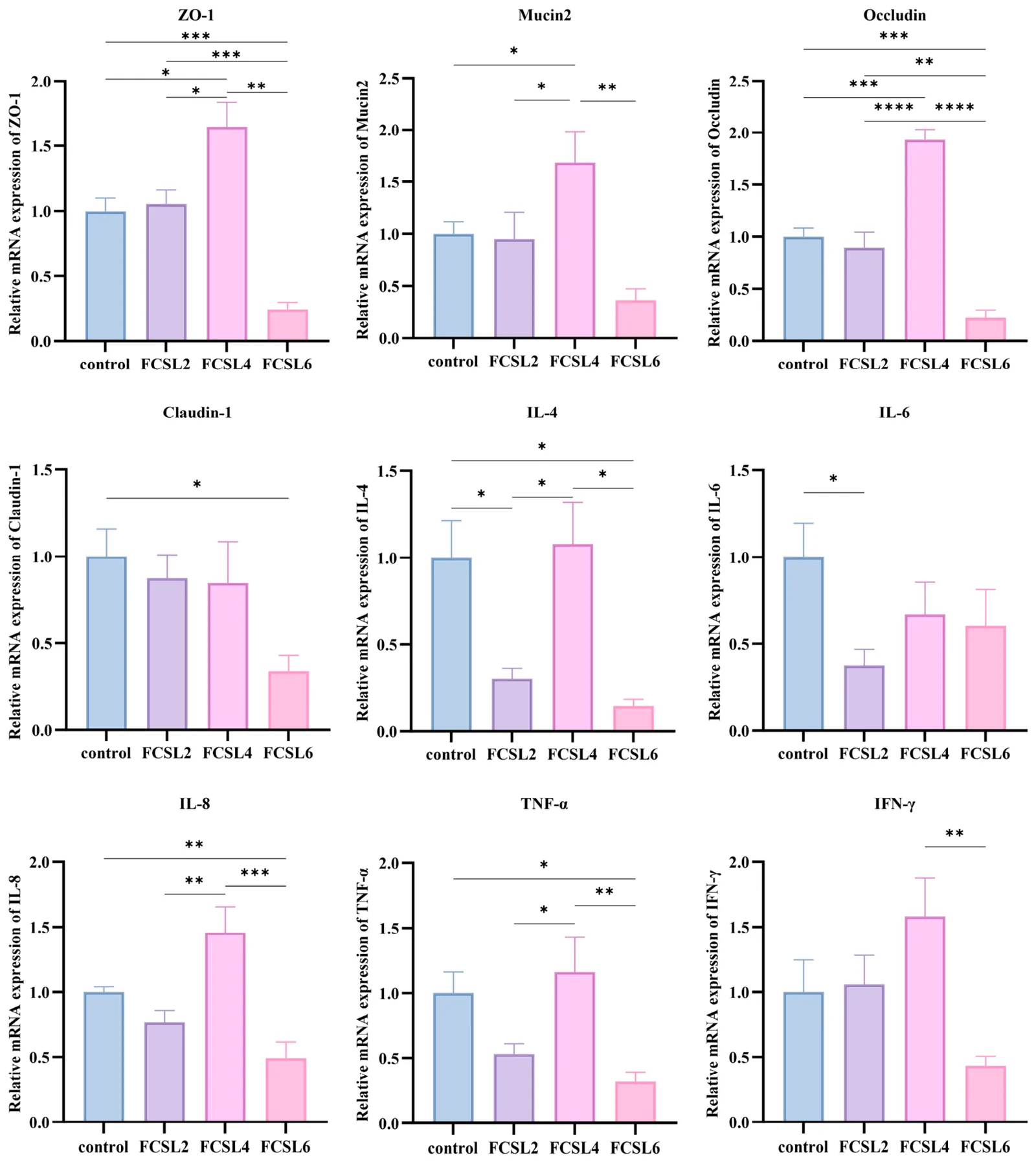
The effects of dietary *Candida utilis*-fermented corn steep liquor on intestinal barrier and immune function mRNA expression in 21-day-old broilers. CU-FCSL2: the experimental diets supplemented with 2% corn steep liquor; CU-FCSL4: the experimental diets supplemented with 4% corn steep liquor; CU-FCSL6: the experimental diets supplemented with 6% corn steep liquor. Bars represent mean ± SME (*n* = 6), * means *p* < 0.05, ** means *p* < 0.01, *** means *p* < 0.005, **** means *p* < 0.001. *n* = 6. ZO-1, zonula occludens-1; IL-4, interleukin-4; IL-6, interleukin-6; IL-8, interleukin-8; TNF-α, tumor necrosis factor-alpha; IFN-γ, interferon γ.

The mRNA expression of tight-junction proteins and inflammatory cytokines in the ileum of 21 d broilers was presented in Figure 1. Real-time quantitative PCR analysis demonstrated that the dietary CU-FCSL inclusion significantly modulated the mRNA expression of tight-junction proteins and inflammatory cytokines in 21 d broilers (*p* < 0.05). The mRNA expression of ZO-1, Occludin-1, and MUC2 was upregulated in the CU-FCSL4 group compared with the other treatments (*p* < 0.05), whereas mostly gene expression was reduced in the CU-FCSL6 group relative to the control (*p* < 0.05). Regarding inflammatory cytokines, IFN-γ, TNF-α, and IL-8 expression was elevated in the CU-FCSL4 group (*p* < 0.05), IL-4 expression was upregulated in the CU-FCSL4 group compared with the CU-FCSL2 and CU-FCSL6 groups (*p* < 0.05), and IL-6 expression was reduced in the CU-FCSL2 group relative to the control (*p* < 0.05). In summary, 4% CU-FCSL enhanced tight-junction protein and MUC2 expression while modulating cytokine profiles, suggesting improved intestinal barrier integrity and immune function, whereas 6% CU-FCSL reduced most gene expression, indicating potential adverse effects at higher inclusion levels.

**Figure 2:**
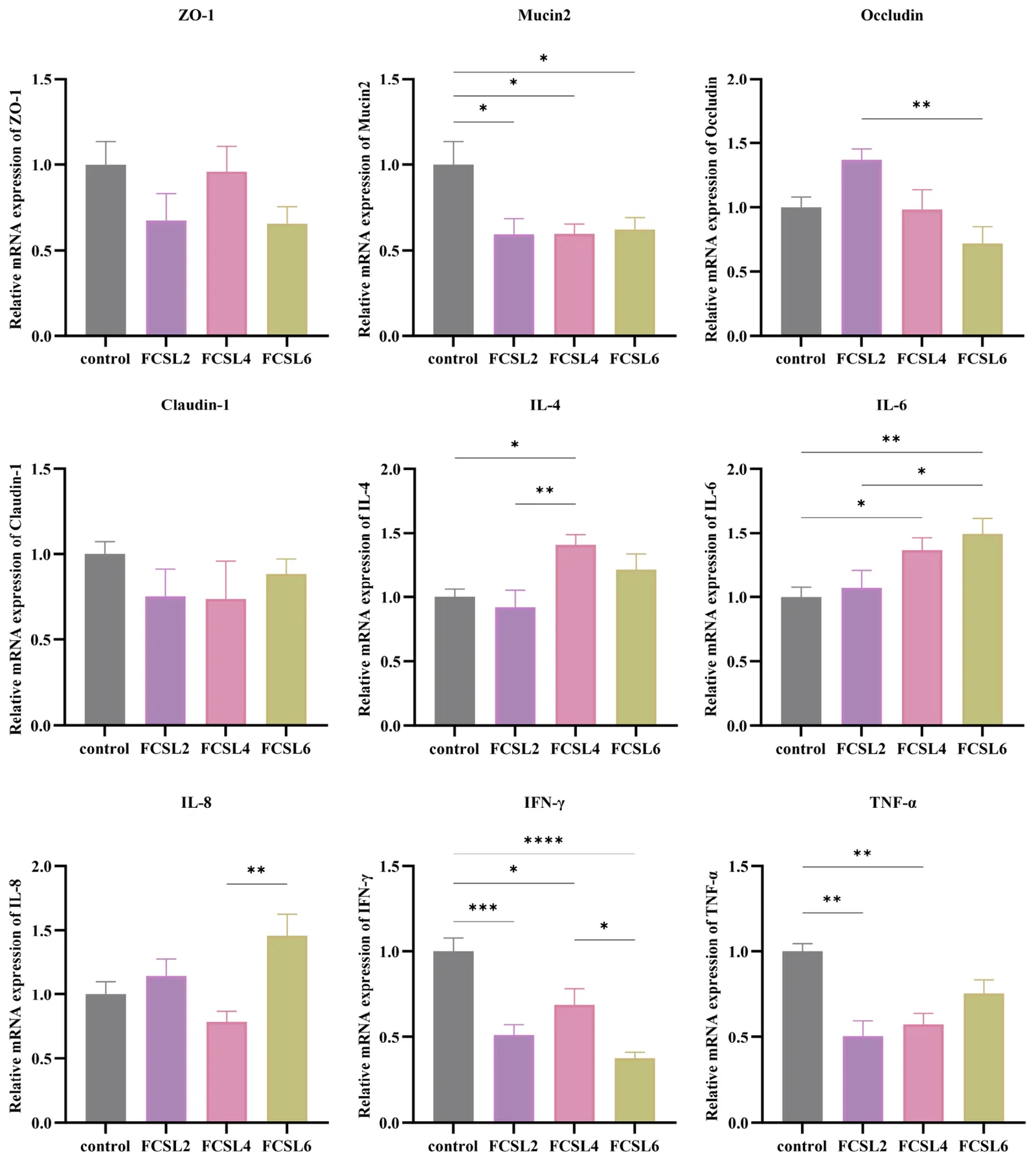
The effects of dietary *Candida utilis*-fermented corn steep liquor on intestinal barrier and immune function mRNA expression in 42 d broilers. CU-FCSL2: the experimental diets supplemented with 2% corn steep liquor; CU-FCSL4: the experimental diets supplemented with 4% corn steep liquor; CU-FCSL6: the experimental diets supplemented with 6% corn steep liquor. Bar represent mean ± SME (*n* = 6), * means *p* < 0.05, ** means *p* < 0.01, *** means *p* < 0.005, **** means *p* < 0.001. *n* = 6. ZO-1, zonula occludens-1; IL-4, interleukin-4; IL-6, interleukin-6; IL-8, interleukin-8; TNF-α, tumor necrosis factor-alpha; IFN-γ, interferon γ.

The mRNA expression of tight-junction proteins and inflammatory cytokines in the ileum of 42 d broilers was presented in Figure 2. No significant differences were detected for ZO-1 and Claudin-1 among treatments. Occludin-1 expression was upregulated in the CU-FCSL2 group relative to the control and the CU-FCSL6 group (*p* < 0.01), while MUC2 expression was reduced in both the CU-FCSL groups compared with the control (*p* < 0.05). Regarding inflammatory cytokines, CU-FCSL supplementation generally upregulated IL-4, IL-6, and IL-8. The CU-FCSL4 group showed elevated IL-4 and IL-6 expression (*p* < 0.05), the CU-FCSL6 group exhibited increased IL-6 and IL-8 expression (*p* < 0.05 and *p* < 0.01, respectively), and the CU-FCSL2 group had no significant changes in these cytokines. By contrast, IFN-γ and TNF-α expression was downregulated in the CU-FCSL2 and CU-FCSL4 groups compared with the control (*p* < 0.001 and *p* < 0.01, respectively).

## 4. Discussion

The nutritional characteristics of CU-FCSL indicated its potential as an alternative protein ingredient for soybean meal in poultry diets. The CP content of 52.82% was slightly above the values commonly reported for conventional corn steep liquor. This ranged from 20–50% depending on processing intensity and source [15,18,26]. The improvement in protein concentration was primarily associated with yeast biomass accumulation during fermentation, as *Candida utilis* (*C. utilis)* generally contained 40–60% protein on a dry matter basis [27,28]. Such protein enrichment effects had also been observed in other water-soluble corn proteins, which were reported to contain CP 52% [18], indicating that microbial fermentation provided an effective strategy for converting low-value agricultural by-products into suitable feed ingredients. Regarding other fermented CSL, He et al. reported a notably lower crude protein content of 26.5% in Saccharomyces cerevisiae-fermented corn steep liquor, a discrepancy that was likely attributed to differences in fermentation substrates, yeast strains, biomass recovery efficiency, and processing conditions. Unlike *S. cerevisiae*, *C. utilis* was characterized by strong adaptability to diverse carbon and nitrogen sources and efficient assimilation of inorganic and organic nitrogen, which may have facilitated microbial protein accumulation during fermentation [28].

The energy evaluation indicated that CU-FCSL provided moderate energy density, with GE of 15.96 MJ/kg on an as-fed basis, AME of 11.36 MJ/kg DM, and AMEn of 11.31 MJ/kg DM. These values were within the range of 9–11 MJ/kg commonly reported for conventional soybean meal [26,29,30], suggesting that fermentation did not compromise the energy availability of the ingredient. Similar trends were reported by He et al., who observed an ME value of 10.78 MJ/kg for *S. cerevisiae*-fermented corn steep liquor in finishing pigs [31]. Nevertheless, experimental groups with 20% CU-FCSL directly replacing the basal diet showed a significant reduction in dietary AME, AMEn, and the AME/GE ratio. Previous studies have reported that the metabolizable energy is positively correlated with crude protein but negatively associated with ash concentrations [32,33]. Since ash has no direct energy contribution and may dilute organic nutrient content [34], the relatively high Ash concentration (18.23%) may partly explain the decreased energy utilization. The elevated ash content was a direct consequence of the sulfur dioxide steeping process during corn wet milling [8] and the enrichment of inorganic salts added during fermentation [10]. Furthermore, the higher nitrogen output was also observed in the CU-FCSL diet, reflecting that the high substitution rate of 20% may have disrupted amino acid balance and resulted in greater catabolism and excretion of surplus nitrogen rather than tissue retention [35].

The amino acid profile and digestibility assessment of CU-FCSL provided critical insights into its protein quality for poultry nutrition. The total amino acid concentration reached 42.38%, with a relatively favorable and balanced indispensable amino acid profile, particularly sulfur-containing amino acids and threonine, which agreed well with that reported by Guo et al. for the soybean meal and water-soluble corn steep liquor protein [18]. The AID and SID values for essential amino acids ranged from 72.67% (tryptophan) to 91.82% (methionine) for AID, and from 82.18% to 94.01% for SID, which aligned closely with high-quality soybean meal [30,36]. He et al., in contrast, reported considerably lower SID values for *S. cerevisiae*-fermented corn steep liquor in finishing pigs: 66.69% for lysine, 66.46% for threonine, and 76.76% for methionine, although different animal species should not be directly compared [31]. The high amino acid digestibility of CU-FCSL may be associated with the high proportion of acid-soluble protein (24.7%) detected in CU-FCSL, which could be rapidly hydrolyzed and absorbed by the gastrointestinal tract. Previous studies have shown that fermentation can partially degrade complex plant proteins into peptides and free amino acids, thereby improving protein accessibility and digestibility [18,20,37]. Guo et al. likewise characterized water-soluble corn protein derived from CSL as being particularly rich in acid-soluble protein [18].

In the present study, the effects of CU-FCSL on growth performance were dependent on inclusion level. Dietary supplementation with 2% CU-FCSL consistently supported optimal growth performance across all phases, and the 4% group maintained performance comparable to the 2% group, whereas 6% CU-FCSL impaired FCR and BWG compared with the control group. These results revealed a relatively narrow effective inclusion bound for CU-FCSL in broiler diets, which differed from that reported for conventional CSL, indicating that the useful inclusion range of CSL-derived ingredients depended strongly on processing and the resulting composition. A recent review article concluded that moderate inclusion levels of 3% to 10% CSL supported growth performance, whereas excessive inclusion exceeding 10% to 15% induced metabolic disturbances [11]. Meanwhile, Yazdanyar et al. observed progressive reductions in feed intake and growth when CSL reached 20–40% in wet-fed broilers. Processed CSL products appear to maintain broiler performance within narrow inclusion ranges, as both a 3% mixture of CSL and enzyme [16], 2.5–5% hydrolyzed CSL [19], and 5% water-soluble corn protein [18] maintained growth performance, whereas higher levels above 10% probably started to diminish feed intake and weight gain. Because the experimental diets were formulated to be isonitrogenous, isocaloric, and balanced for key digestible amino acids, the variations in growth performance were unlikely to arise from the basic nutritional values of the diet. The narrower effective range from 2% to 4% observed in the present study likely embodied the concentrated nature of CU-FCSL as a fermented product, where yeast biomass proliferation has simultaneously elevated protein content and accumulated non-nutritive components such as minerals, nucleic acids, and fumonisins. Particularly, the total fumonisins in CU-FCSL was 29.70 mg/kg, giving an estimated 6% complete-feed contribution of 1.78 mg/kg. Because the EU recommendation for fumonisins was defined as the sum of FB_1_ and FB_2_, the relevant concentration was 22.98 mg/kg, which was below the guidance value for maize products of 40 mg/kg, while the calculated contribution at a 6% dietary inclusion to the complete feed reached approximately 1.38 mg/kg, approaching the newest EU poultry formulated feed limit of 2 mg/kg [38], which indicated safety considerations in higher inclusion. The low-level inclusion group supplied favorable protein quality, high amino acid availability, and fermentation-derived functional components that influenced nutrient utilization and intestinal development, without introducing excessive amounts of accompanying non-protein components that may limit efficiency [39,40]. The balance between nutritional and limiting factors must be considered when using fermented ingredients in the diet.

Carcass characteristics further supported the suitability of CU-FCSL as a partial soybean meal replacement ingredient. In the present study, CU-FCSL supplementation did not adversely affect most slaughter parameters, indicating that a 2–6% inclusion level maintained overall carcass development, in line with recent studies showing that 5% CSL-derived WCP maintained most carcass traits and increased dressing percentage [18]. However, the increased abdominal fat at the highest CU-FCSL level suggested a possible shift in nutrient partitioning. When absorbed amino acids and available energy did not optimally balance, or when additional metabolic costs were incurred in handling nitrogen and mineral loads, less retained energy may be directed toward protein accretion and more toward lipid deposition [41,42].

The results of CU-FCSL on nutrient digestibility showed that 2% CU-FCSL reduced apparent CP, OM, and EE digestibility at 21 d, and by 42 d of age, the differences in OM and EE digestibility had disappeared, while CP digestibility was greatest in birds receiving 6% CU-FCSL. This pattern indicated that nutrient digestibility responded differently across inclusion levels and growth stages, a phenomenon also observed in previous studies on conventional CSL, which found that in isonitrogenous and isocaloric broiler diets, 5% CSL resulted in the highest DM digestibility during the starter phase, 10% produced the highest CP digestibility, and CP digestibility during the finisher phase was greater at 5% and 15% than in the control [15]. In contrast, Hajati et al. reported that CP digestibility remained unchanged at 2.5–5% hydrolyzed CSL but declined at 10%, indicating that the digestive response depends not only on dosage but also on processing and product composition [19]. The relatively high CP digestibility observed with 6% CU-FCSL at 42 d may reflect the changes in protein composition generated during *C. utilis* fermentation and a better capacity to utilize digestive enzymes improved with age [10,39,43]. But the advantage did not translate into superior growth, while the 2% group achieved the most favorable productive response despite lower apparent digestibility at 21 d. This divergence indicates that the feeding value of CU-FCSL cannot be judged solely by apparent nutrient disappearance. A more reliable explanation is that postabsorptive amino acid utilization, energy efficiency, and the accompanying residual mycotoxin, mineral, and purine load may become more important than digestion alone at higher inclusion levels.

Intestinal morphology and gene expression revealed that the beneficial effects of CU-FCSL did not entirely parallel growth performance, suggesting a specific role for fermentation-derived components in regulating intestinal development. The 4% CU-FCSL treatment produced the most favorable intestinal morphology, with a higher VCR, indicating a beneficial absorptive capacity, but increasing CU-FCSL to 6% provided no further morphological advantage. This trend aligned with recent findings that low HCSL inclusion exhibited greater jejunal morphology, but these benefits diminished as dietary inclusion increased to 10% [19]. Intestinal gene expression provided further insight into the molecular response to CU-FCSL supplementation. At 21 d, 4% CU-FCSL markedly upregulated the barrier-related genes ZO-1, Occludin-1, and MUC2, while IFN-γ, TNF-α, IL-8, and IL-4 were also increased, suggesting that reinforcement of epithelial barrier function occurred together with modulation of mucosal immune signaling. A modified mucosal immune transcriptional profile was observed by 42 d, although the present data did not establish a sequential mechanism of immune maturation. The 2% CU-FCSL maintained higher MUC2 and Occludin-1 expression; all CU-FCSL treatments reduced IFN-γ, and 2% and 4% also reduced TNF-α relative to the control, while 4% retained higher IL-4 and IL-6. Rather than indicating generalized immune suppression, this shift suggests maturation of intestinal immunity and adaptation to prolonged dietary exposure. Barrier-related signaling therefore remained active while several pro-inflammatory pathways were attenuated. Evidence directly linking fermented corn-derived ingredients with intestinal barrier gene expression in broilers remains limited. Fermented feed products could regulate tight-junction and MUC2 expression even after microbial inactivation, suggesting that fermentation-derived metabolites may contribute to barrier function even without viable microorganisms. Similar responses have been reported with fermented feed ingredients [44]. The intestinal benefits of CU-FCSL likely resulted from the combined effects of nutrient characteristics and the components generated during *Candida utilis* fermentation. The high glutamic acid content of CU-FCSL (8.19%) may provide an additional nutritional advantage because intestinal tissues extensively utilize glutamate, which contributes to epithelial energy metabolism [45]. Fermentation increased the proportion of soluble nitrogen compounds and acid-soluble protein, providing small peptides and amino acids that could partly support intestinal epithelial turnover and regulate intestinal development [39,46]. In addition to improved nutrient accessibility, yeast fermentation products may directly influence intestinal barrier function. The cell-wall components of yeast, particularly β-glucans and mannan oligosaccharides, have been reported to influence villus development, epithelial integrity, and epithelial immune signaling [47]. The presence of approximately 3% mannan in CU-FCSL may explain the improved intestinal structure, although its specific structural form and biological activity were not characterized in the present study. The morphological and transcriptional findings suggested that an intermediate CU-FCSL inclusion level may provide the most favorable balance between nutrient availability and fermentation-derived functional components, while higher inclusion did not necessarily result in further intestinal benefit. Further studies integrating intestinal protein expression, mucosal immune cell profiling, and microbial or metabolomic responses are needed to clarify the mechanisms underlying these effects, which depended on age and dose.

Serum biochemical and immune parameters showed that 2% and 4% CU-FCSL generally supported a more favorable balance between serum humoral immunity and metabolism, while the highest inclusion introduced a greater burden of nitrogen metabolism associated with purines. Notably, UA concentration was significantly elevated in the 6% group at both 21 and 42 days, whereas the 2% and 4% groups remained comparable to the control. This finding aligned with earlier observations that serum UA tended to increase progressively as dietary CSL increased from 0 to 15% [15], and the recent review by Lee et al. likewise noted that excessive CSL inclusion was linked to nephrotoxic effects caused by elevated serum uric acid [11]. After purines and nucleic acids were catabolized in broilers, they were ultimately excreted primarily in the form of UA due to the absence of arginase in the liver [48]. In the present study, CU-FCSL contained 4.89 g/kg total purines, mainly hypoxanthine and guanine, directly associated with the elevation in UA. At low dietary CU-FCSL inclusion, renal excretion capacity maintained normal UA concentrations, while purine intake exceeded renal clearance capacity at 6% inclusion. This metabolic burden may partially explain the higher nitrogen output observed at 20% inclusion in the energy trial and the failure of greater CP digestibility at 42 d to translate into superior growth, as energy and nutrients were diverted from growth to uric acid synthesis and excretion. By comparison, Guo et al. found that 5% CSL-derived water-soluble corn protein maintained ALT, AST, and BUN within the control range, indicating that extensively processed CSL proteins may support protein metabolism [18]. The serum immune parameters further illustrated the complex relationship between inclusion and performance. The 2% inclusion showed the most favorable immune profile overall, with lower CRP and higher IgG, IgA, and IgM, indicating enhanced humoral immunity without disrupting immune balance. In contrast, the 4% inclusion paradoxically elevated both IL-10 and CRP, revealing a stress-inducing potential despite the anti-inflammatory signal, and the 6% inclusion severely impaired immune balance. Ebrahimpour et al. reported that 6% dried CSL enhanced antibody titers against Newcastle disease, influenza, and bronchitis viruses in broilers, demonstrating that moderate to high levels can stimulate humoral immunity, though the optimal level may vary by immune parameter and challenge status [17]. Fermented feeds and yeast products could supply small peptides, organic acids, mannans, and other cell-wall fractions that influence B-cell activity, mucosal signaling, and innate immune recognition rather than simply producing uniform immune stimulation [27,39]. Changes in immune organ development were modest but directionally consistent with this immunomodulatory response.

## 5. Conclusions

The present study establishes that CU-FCSL was a nutritionally valuable alternative protein source for broilers, characterized by an AME of 11.36 MJ/kg DM and high SID values for essential amino acids ranging from 82.18% (tryptophan) to 94.01% (methionine). In the 42-day feeding trial, dietary inclusion of CU-FCSL at 2% and 4% significantly improved final body weight and overall weight gain compared with the control, and without adverse effects on nutrient digestibility, serum immune indices, and intestinal health. Conversely, 6% inclusion negatively affected growth performance, increased abdominal fat deposition and drip loss, elevated serum uric acid and ALT, reduced intestinal villus-to-crypt ratio, and compromised humoral immune parameters. These findings indicated that 2% and 4% CU-FCSL could be added to effectively replace SBM; excessive inclusion may induce metabolic demand and compromise intestinal integrity, likely due to the accumulation of yeast metabolites, ash, purines, or residual anti-nutritional factors. Future research should focus on optimizing fermentation and detoxification processes to reduce purine content and anti-nutritional factors.

## Figures and Tables

**Table 1:** The chemical composition and amino acid profile of *Candida utilis*-fermented corn steep liquor (as-fed basis).

Item	Value	Item	Value
Conventional nutrients		Total 18 amino acids, %	42.38
Dry matter, %	88.01	Essential amino acids	
Crude protein, %	52.82	Arg, %	2.14
Acid-soluble protein, %	24.7	His, %	1.51
Ether extract, %	<1.00	Ile, %	1.41
Crude fiber, %	5.2	Leu, %	3.06
Ash, %	18.23	Lys, %	1.93
Calcium, %	0.053	Met, %	0.7
Total phosphorus, %	2.25	Phe, %	1.12
Phytate phosphorus, %	0.46	Thr, %	2.28
Potassium, %	5.4	Trp, %	0.23
Sodium, %	0.58	Val, %	2.18
Chlorine, %	0.93	Non-essential amino acids	
Gross energy, MJ/kg	15.96	Ala, %	3.35
Other components		Asp, %	4.74
Fumonisin B1, mg/kg	22.25	Cys, %	1.09
Fumonisin B2, mg/kg	0.73	Glu, %	8.19
Fumonisin B3, mg/kg	6.72	Gly, %	2.58
Zearalenone, μg/kg	7.2	Pro, %	2.97
T-2 toxin, ug/kg	1.97	Ser, %	2.26
Total acid, g/kg	4.7	Tyr, %	1.24
Mannan, %	3		
Total purine, g/kg	4.89		
Hypoxanthine, g/kg	2.46		
Guanine, g/kg	1.65		
Adenine, g/kg	0.64		
Xanthine, g/kg	0.14		

**Table 2:** Ingredient composition and calculated nutrient levels of the basal diet in Exp. 1 and Exp. 2 (as-fed basis).

Ingredient	Inclusion (%)	Calculated Nutrient	Value
Corn	64.8	GE (MJ/kg)	17.35
Soybean meal	19.7	AME (MJ/kg)	13.61
Corn gluten meal	2.1	DM (%)	87.75
Wheat flour	5.0	CP (%)	17.71
Soybean oil	3.2	Ca (%)	0.67
Limestone	1.0	Non-phytate P (%)	0.28
NaCl	0.15	Digestible Lys (%)	1.08
Dicalcium phosphate	1.0	Digestible Met (%)	0.63
NaHCO_3_	0.28	Digestible Met + Cys (%)	0.86
Choline chloride (50%)	0.10	Digestible Thr (%)	0.72
L-Lys·HCl (98%)	0.56		
DL-Met (98%)	0.40		
L-Thr (98.5%)	0.25		
L-Arg (98%)	0.30		
L-Trp (98%)	0.03		
L-Val (98%)	0.19		
L-Ile (98.5%)	0.18		
TiO_2_	0.50		
Vitamin premix ^1^	0.02		
Mineral premix ^2^	0.20		
Phytase (10,000 U/g)	0.02		
Antioxidant	0.02		
Total	100		

^1^ The vitamin premix provided (per kg of diets) the following: vitamin A, 8000 IU; vitamin D_3_, 2500 IU; vitamin E, 30 IU; vitamin K_3_, 2 mg; vitaminB_1_, 1.60 mg; vitaminB_2_, 5 mg; vitaminB_6_, 3 mg; vitamin B_12_, 0.02 mg; *D*-biotin, 0.12 mg; *D*-pantothenic acid, 9 mg; folic acid, 1 mg; nicotinamide, 32 mg; Ethoxyquin, 1 mg. ^2^ The trace mineral premix provided (per kg of diets) the following: Fe, 100 mg; Cu, 12 mg; Mn, 100 mg; Zn, 80 mg; Se, 0.35 mg; I, 1.5 mg.

**Table 3:** Composition of the nitrogen-free diet.

Ingredient	Inclusion (%)
Glucose	22.00
Corn starch	39.00
Pea starch	28.00
Wood cellulose fiber	4.00
Soybean oil	3.00
Dicalcium phosphate	1.80
Limestone	1.00
Sodium chloride	0.30
Choline chloride (50%)	0.18
Vitamin premix ^1^	0.02
Mineral premix ^2^	0.20
TiO_2_	0.50
Total	100.00

^1^ The vitamin premix provided (per kg of diets) the following: vitamin A, 8000 IU; vitamin D_3_, 2500 IU; vitamin E, 30 IU; vitamin K_3_, 2 mg; vitaminB_1_, 1.60 mg; vitaminB_2_, 5 mg; vitaminB_6_, 3 mg; vitamin B_12_, 0.02 mg; *D*-biotin, 0.12 mg; *D*-pantothenic acid, 9 mg; folic acid, 1 mg; nicotinamide, 32 mg; Ethoxyquin, 1 mg. ^2^ The trace mineral premix provided (per kg of diets) the following: Fe, 100 mg; Cu, 12 mg; Mn, 100 mg; Zn, 80 mg; Se, 0.35 mg; I, 1.5 mg.

**Table 4:** Ingredient composition and calculated nutrient levels of the basal diet and experimental diet of starter (1–21 d) phases in Exp. 3.

Ingredient (%)	Basal Diet	Experimental Diet of CU-FCSL		
		2%	4%	6%
Corn	55.73	56.49	57.24	58.01
Soybean meal	25.25	22.8	20.33	17.88
Corn gluten meal	5	5	5	5
Wheat flour	5	5	5	5
Soybean oil	3.46	3.13	2.81	2.48
Limestone	1.15	1.19	1.23	1.27
NaCl	0.25	0.25	0.25	0.25
Dicalcium phosphate	1.25	1.21	1.17	1.13
NaHCO_3_	0.2	0.2	0.2	0.2
Choline chloride (50%)	0.1	0.1	0.1	0.1
L-Lys·HCl (98%)	0.39	0.63	0.65	0.66
DL-Met (98%)	0.3	0.4	0.39	0.38
L-Thr (98.5%)	0.07	0.29	0.29	0.28
L-Try (98%)	0.32	0.07	0.08	0.08
L-Arg (98%)	0.16	0.31	0.33	0.35
L-Val (98%)	0.39	0.17	0.17	0.17
TiO_2_	0.5	0.5	0.5	0.5
Vitamin premix ^1^	0.02	0.02	0.02	0.02
Mineral premix ^2^	0.2	0.2	0.2	0.2
Phytase (10,000 U/g)	0.02	0.02	0.02	0.02
Antioxidant	0.02	0.02	0.02	0.02
CU-FCSL	0	2	4	6
Total	100	100	100	100
AME, Mcal/kg	2.98	2.98	2.98	2.98
CP, %	21.49	21.49	21.51	21.51
Ga, %	0.8	0.8	0.8	0.8
Non-phytate P, %	0.33	0.33	0.33	0.33
Digestible Lys, %	1.26	1.26	1.26	1.26
Digestible Met + Cys, %	0.95	0.95	0.95	0.95
Digestible Thr, %	0.87	0.87	0.87	0.87
Digestible Val, %	0.96	0.96	0.96	0.96
Digestible Arg, %	1.34	1.34	1.34	1.34
Digestible Try, %	0.22	0.22	0.22	0.22

^1^ The vitamin premix provided (per kg of diets) the following: vitamin A, 8000 IU; vitamin D_3_, 2500 IU; vitamin E, 30 IU; vitamin K_3_, 2 mg; vitaminB_1_, 1.60 mg; vitaminB_2_, 5 mg; vitaminB_6_, 3 mg; vitamin B_12_, 0.02 mg; *D*-biotin, 0.12 mg; *D*-pantothenic acid, 9 mg; folic acid, 1 mg; nicotinamide, 32 mg; Ethoxyquin, 1 mg. ^2^ The trace mineral premix provided (per kg of diets) the following: Fe, 100 mg; Cu, 12 mg; Mn, 100 mg; Zn, 80 mg; Se, 0.35 mg; I, 1.5 mg.

**Table 5:** Ingredient composition and calculated nutrient levels of the basal diet and experimental diet of the growth (21–42 d) phases in Exp. 3.

Ingredient	Basal Diet	Experimental Diet of CU-FCSL		
		2%	4%	6%
Corn	61.70	63.40	69.52	69.32
Soybean meal	17.58	17.54	13.12	12.16
Corn gluten meal	4.12	3.27	5.00	4.12
Wheat flour	8.00	5.00		
Soybean oil	2.88	3.15	2.52	2.56
Limestone	0.95	0.97	1.00	1.03
NaCl	0.25	0.25	0.25	0.25
Dicalcium phosphate	1.35	1.32	1.32	1.28
NaHCO_3_	0.20	0.20	0.20	0.20
Choline chloride (50%)	0.10	0.10	0.10	0.10
L-Lys·HCl (98%)	0.71	0.67	0.74	0.73
DL-Met (98%)	0.36	0.36	0.35	0.36
L-Thr (98.5%)	0.25	0.23	0.24	0.23
L-Try (98%)	0.04	0.04	0.06	0.06
L-Arg (98%)	0.40	0.38	0.45	0.44
L-Val (98%)	0.17	0.17	0.16	0.17
TiO_2_	0.50	0.50	0.50	0.50
Vitamin premix ^1^	0.02	0.02	0.02	0.02
Mineral premix ^2^	0.20	0.20	0.20	0.20
Phytase (10,000 U/g)	0.02	0.02	0.02	0.02
Antioxidant	0.02	0.02	0.02	0.02
CU-FCSL	0.00	2.00	4.00	6.00
Total	100	100	100	100
AME, Mcal/kg	3.18	3.18	3.18	3.18
CP, %	18.49	18.50	18.50	18.50
Ga, %	0.72	0.73	0.74	0.74
Non-phytate P, %	0.31	0.31	0.31	0.31
Digestible Lys, %	1.15	1.15	1.15	1.15
Digestible Met + Cys, %	0.84	0.84	0.84	0.85
Digestible Thr, %	0.74	0.74	0.74	0.74
Digestible Val, %	0.87	0.87	0.87	0.87
Digestible Arg, %	1.21	1.21	1.21	1.21
Digestible Try, %	0.18	0.18	0.18	0.18

^1^ The vitamin premix provided (per kg of diets) the following: vitamin A, 8000 IU; vitamin D_3_, 2500 IU; vitamin E, 30 IU; vitamin K_3_, 2 mg; vitaminB_1_, 1.60 mg; vitaminB_2_, 5 mg; vitaminB_6_, 3 mg; vitamin B_12_, 0.02 mg; *D*-biotin, 0.12 mg; *D*-pantothenic acid, 9 mg; folic acid, 1 mg; nicotinamide, 32 mg; Ethoxyquin, 1 mg. ^2^ The trace mineral premix provided (per kg of diets) the following: Fe, 100 mg; Cu, 12 mg; Mn, 100 mg; Zn, 80 mg; Se, 0.35 mg; I, 1.5 mg.Animal Management and Sample Collection.

**Table 6:** Primer sequences for RT-PCR.

Gene	Forward Sequences (5′-3′)	Reverse Sequences (5′-3′)
*β-actin*	GAGAAATTGTGCGTGACATCA	CCTGAACCTCTCATTGCCA
*ZO-1*	GGATGTTTATTTGGGCGGCT	CCATTGTTGCACTCTTGCCG
*Occludin* *-1*	ACGGCAGCACCTACCTCAA	GGGCGAAGAAGCAGATGAG
*M* *UC* *2*	CTGCTGTGCTCCACCATTAAGTCC	GCTTGACACGCTCGGAGTATAACG
*Claudin-1*	CATACTCCTGGGTCTGGTTGGT	GGGCGAAGAAGCAGATGAG
*TNF-α*	CCCCTACCCTGTCCCACAA	TGAGTACTGCGGAGGGTTCAT
*IFN-γ*	AAAGCCGCACATCAAACACA	GCCATCAGGAAGGTTGTTTTTC
*IL-6*	CGCCCAGAAATCCCTCCTC	AGGCACTGAAACTCCTGGTC
*IL-8*	GGCTTGCTAGGGGAAATGA	AGCTGACTCTGACTAGGAAACTGT
*IL-4*	AGCACTGCCACAAGAACCTG	CCTGCTGCCGTGGGACAT

*ZO-1*, zonula occludens-1; *IL-4*, interleukin-4; *IL-6*, interleukin-6; *IL-8*, interleukin-8; *TNF-α*, tumor necrosis factor-alpha; *IFN-γ*, interferon γ.

**Table 7:** The effects of dietary CU-FCSL inclusion on energy utilization and nitrogen balance.

Items	Basal Diet	20% CU-FCSL	SEM	*p*-Value
Nitrogen balance (g/d)				
Nitrogen intake	3.39	3.70	0.18	0.73
Nitrogen output	1.60	2.00	0.09	0.037
Nitrogen retained	1.79	1.70	0.13	0.94
Available energy/(MJ/kg DM)				
AME	13.61	13.15	0.20	0.028
AMEn	13.55	13.09	0.20	0.027
Energy conversion efficiency/%				
AME/GE	78.42	73.83	0.93	0.039

CU-FCSL, *Candida utilis*-fermented corn steep liquor; AME, apparent metabolizable energy; AMEn, apparent metabolizable energy corrected for nitrogen; SEM, standard error of the mean.

**Table 8:** Apparent and standardized ileal digestibility of amino acids in CU-FCSL for broilers (%).

Amino Acid	Endogenous Loss (mg/kg DM)	AID	SID
Essential amino acids			
Arg	577.62	89.21	92.65
His	410.75	84.17	87.53
Ile	605.41	83.79	88.93
Leu	1090.84	86.29	90.73
Lys	795.26	85.15	90.00
Met	188.55	91.82	94.01
Phe	648.24	84.64	89.88
Thr	964.86	78.80	84.63
Trp	164.81	72.67	82.18
Val	863.21	83.92	88.86
Non-essential amino acids			
Ala	876.49	85.15	88.69
Asp	1640.17	80.33	84.96
Cys	608.82	65.66	72.37
Glu	2141.27	77.89	89.14
Gly	857.43	76.55	80.92
Pro	1029.45	78.92	84.27
Ser	893.63	78.52	84.41
Tyr	311.10	87.65	89.57

AID: apparent ileal digestibility; SID: standardized ileal digestibility; Ala, alanine; Arg, arginine; Asp, aspartic acid; Cys, cysteine; Glu, glutamic acid; Gly, glycine; His, histidine; Ile, isoleucine; Leu, leucine; Lys, lysine; Met, methionine; Phe, phenylalanine; Pro, proline; Ser, serine; Thr, threonine; Trp, tryptophan; Tyr, tyrosine; Val, valine.

**Table 9:** Effects of *Candida utilis*-fermented corn steep liquor on growth performance of broilers.

	Starter Phase (1–21 d)					Finisher Phase (22–42 d)				Whole Period (1–42 d)		
Group	1 d BW, kg	21 d BW, kg	BWG, kg	FI, kg	FCR	42 d BW, kg	BWG, kg	FI, kg	FCR	BWG, kg	FI, kg	FCR
control	0.04	0.862	0.822	1.11	1.36 ^b^	2.58 ^b^	1.71 ^b^	2.93	1.68	2.54 ^b^	3.79 ^b^	1.47 ^b^
CU-FCSL2	0.04	0.881	0.841	1.04	1.24 ^c^	2.80 ^a^	1.91 ^a^	3.02	1.66	2.76 ^a^	4.11 ^a^	1.48 ^b^
CU-FCSL4	0.04	0.861	0.821	1.1	1.34 ^bc^	2.75 ^a^	1.89 ^a^	3.03	1.68	2.71 ^a^	4.13 ^a^	1.52 ^ab^
CU-FCSL6	0.04	0.800	0.760	1.09	1.43 ^a^	2.61 ^b^	1.83 ^a^	3.03	1.72	2.57 ^b^	4.03 ^ab^	1.57 ^a^
SEM	0.042	0.011	0.011	0.015	0.045	0.029	0.024	0.025	0.016	0.029	0.052	0.064
*p*-value	0.111	0.072	0.072	0.366	0.005	0.008	0.008	0.507	0.488	0.008	0.048	0.013

^a–c^ Means within a row with different superscripts differ significantly (*p* < 0.05). CU-FCSL2: the experimental diets supplemented with 2% corn steep liquor; CU-FCSL4: the experimental diets supplemented with 4% corn steep liquor; CU-FCSL6: the experimental diets supplemented with 6% corn steep liquor; SEM, standard error of the mean; BW, body weight; BWG, body weight gain; FI, feed intake; FCR, feed conversion ratio.

**Table 10:** Effect of *Candida utilis*-fermented corn steep liquor on carcass traits and meat quality of 42-day-old broilers (%).

Group	Carcass Rate	Half-Eviscerated Rate	Eviscerated Rate	Breast Muscle Rate	Thigh Rate	Abdominal Fat Rate	Drip Loss
control	92.06	88.61	75.78	27.51	19.70	1.46 ^b^	2.41 ^b^
CU-FCSL2	93.79	89.19	75.10	30.23	19.56	2.18 ^a^	2.61 ^b^
CU-FCSL4	93.52	88.54	75.74	33.90	19.37	1.42 ^b^	2.64 ^b^
CU-FCSL6	94.07	89.18	76.67	32.84	19.54	2.24 ^a^	3.31 ^a^
SEM	0.21	0.79	1.14	0.54	1.47	0.086	0.097
*p*-value	0.091	0.148	0.665	0.261	0.444	0.029	0.044

^a–b^ Means within a row with different superscripts differ significantly (*p* < 0.05). CU-FCSL2: the experimental diets supplemented with 2% corn steep liquor; CU-FCSL4: the experimental diets supplemented with 4% corn steep liquor; CU-FCSL6: the experimental diets supplemented with 6% corn steep liquor; SEM, standard error of the mean.

**Table 11:** Effect of *Candida utilis*-fermented corn steep liquor on immune organ indices of 21- and 42-day-old broilers (g/kg).

Group	21-Day-Old				42-Day-Old			
	Liver	Spleen	Bursa of Fabricius	Thymus	Liver	Spleen	Bursa of Fabricius	Thymus
control	33.19	1.04 ^b^	1.94	8.12	27.44	1.29	0.41	5.27 ^c^
CU-FCSL2	33.58	1.07 ^b^	1.78	6.56	25.42	1.36	0.38	5.68 ^bc^
CU-FCSL4	31.89	1.35 ^a^	1.81	6.99	28.68	1.36	0.40	7.76 ^ab^
CU-FCSL6	32.67	1.08 ^b^	1.92	7.63	29.09	1.25	0.37	8.74 ^a^
SEM	2.48	0.18	0.34	1.37	3.82	0.19	0.1	2.31
*p*-value	0.729	0.031	0.346	0.32	0.548	0.721	0.934	0.015

^a–c^ Means within a row with different superscripts differ significantly (*p* < 0.05). CU-FCSL2: the experimental diets supplemented with 2% corn steep liquor; CU-FCSL4: the experimental diets supplemented with 4% corn steep liquor; CU-FCSL6: the experimental diets supplemented with 6% corn steep liquor; SEM, standard error of the mean.

**Table 12:** Effect of *Candida utilis*-fermented corn steep liquor on intestinal morphology parameters of 21- and 42-day-old broilers.

Group	21-Day-Old			42-Day-Old		
	VH, μm	CD, μm	VCR	VH, μm	CD, μm	VCR
control	1000.89 ^c^	164.92 ^b^	6.17 ^a^	1094.65	170.48 ^ab^	6.75 ^b^
CU-FCSL2	1163.36	196.08 ^a^	6.27 ^a^	1218.06	197.05 ^a^	6.36 ^b^
CU-FCSL4	1218.50	190.84 ^a^	6.58 ^a^	1121.43	147.89 ^b^	7.88 ^a^
CU-FCSL6	1062.52	213.14 ^a^	5.48 ^b^	1109.71	189.63 ^a^	5.86 ^c^
SEM	28.21	10.41	0.27	32.70	5.78	0.23
*p*-value	0.511	0.031	0.037	0.607	0.005	0.025

^a–c^ Means within a row with different superscripts differ significantly (*p* < 0.05). CU-FCSL2: the experimental diets supplemented with 2% corn steep liquor; CU-FCSL4: the experimental diets supplemented with 4% corn steep liquor; CU-FCSL6: the experimental diets supplemented with 6% corn steep liquor; SEM, standard error of the mean; VH, villus height; CD, crypt depth; VCR, the ratio of villus height-to-crypt depth.

**Table 13:** Effect of *Candida utilis*-fermented corn steep liquor on nutrient digestibility of 21- and 42-day-old broilers (%).

Group	21-Day-Old			42-Day-Old		
	Crude Protein	Organic Matter	Ether Extract	Crude Protein	Organic Matter	Ether Extract
control	64.66 ^a^	73.13 ^a^	72.60 ^bc^	59.56 ^b^	80.60	82.82
CU-FCSL2	56.21 ^b^	67.79 ^b^	68.41 ^c^	58.92 ^b^	79.99	83.24
CU-FCSL4	66.26 ^a^	73.68 ^a^	79.40 ^a^	58.69 ^b^	80.72	83.49
CU-FCSL6	63.08 ^a^	74.56 ^a^	75.79 ^ab^	65.55 ^a^	81.03	84.69
SEM	1.09	0.67	1.33	1.09	0.49	0.49
*p*-value	0.006	0.001	0.008	0.021	0.910	0.598

^a–c^ Means within a row with different superscripts differ significantly (*p* < 0.05). CU-FCSL2: the experimental diets supplemented with 2% corn steep liquor; CU-FCSL4: the experimental diets supplemented with 4% corn steep liquor; CU-FCSL6: the experimental diets supplemented with 6% corn steep liquor; SEM, standard error of the mean.

**Table 14:** Effect of *Candida utilis*-fermented corn steep liquor on serum biochemistry of 21- and 42-day-old broilers.

Group	21-Day-Old				42-Day-Old			
	ALT (U/L)	AST (U/L)	BUN (mmol/L)	UA (umol/L)	ALT (U/L)	AST (U/L)	BUN (mmol/L)	UA (umol/L)
control	1.33 ^b^	419.50	0.62	265.17 ^c^	18.67	281.67	2.47 ^a^	529.83 ^a^
CU-FCSL2	2.33 ^a^	523.17	0.68	329.67 ^ab^	20.17	265.50	2.17 ^a^	432.50 ^ab^
CU-FCSL4	1.50 ^b^	483.17	0.65	285.8 ^bc^	23.67	298.00	1.68 ^b^	360.00 ^b^
CU-FCSL6	2.17 ^a^	450.00	0.63	399.5 ^a^	21.83	235.17	2.52 ^a^	535.17 ^a^
SEM	0.16	19.89	0.03	16.81	0.78	5.69	0.08	22.77
*p*-value	0.026	0.651	0.562	0.014	0.375	0.29	0.013	0.038

^a–c^ Means within a row with different superscripts differ significantly (*p* < 0.05). CU-FCSL2: the experimental diets supplemented with 2% corn steep liquor; CU-FCSL4: the experimental diets supplemented with 4% corn steep liquor; CU-FCSL6: the experimental diets supplemented with 6% corn steep liquor; SEM, standard error of the mean; ALT, alanine aminotransferase; AST, aspartate aminotransferase; BUN, blood urea nitrogen; UA, uric acid.

**Table 15:** The effects of dietary *Candida utilis*-fermented corn steep liquor on serum immune parameters in 21-day-old broilers.

Group	IgG (mg/mL)	IgA (ug/mL)	IgM (ug/mL)	IL6 (pg/mL)	IL10 (pg/mL)	CRP (pg/mL)
control	16.10 ^a^	109.26	197.47 ^ab^	17.18	20.70 ^b^	35.89 ^b^
CU-FCSL2	16.69 ^a^	100.9	252.20 ^a^	19.16	19.58 ^b^	18.07 ^c^
CU-FCSL4	17.66 ^a^	94.87	142.35 ^b^	26.43	32.74 ^a^	69.77 ^a^
CU-FCSL6	11.86 ^b^	107.77	132.17 ^b^	15.10	18.34 ^b^	33.90 ^b^
SEM	0.69	11.34	13.73	2.55	3.21	3.08
*p*-value	0.011	0.394	0.036	0.108	0.033	0.024

^a–c^ Means within a row with different superscripts differ significantly (*p* < 0.05). CU-FCSL2: the experimental diets supplemented with 2% corn steep liquor; CU-FCSL4: the experimental diets supplemented with 4% corn steep liquor; CU-FCSL6: the experimental diets supplemented with 6% corn steep liquor; SEM, standard error of the mean.

**Table 16:** The effects of dietary *Candida utilis*-fermented corn steep liquor on serum immune parameters in 42-day-old broilers.

Group	IgG (mg/mL)	IgA (ug/mL)	IgM (ug/mL)	IL6 (pg/mL)	IL10 (pg/mL)	CRP (pg/mL)
control	2.95 ^b^	48.57 ^b^	90.26 ^b^	66.68 ^b^	171.32	145.06
CU-FCSL2	5.86 ^a^	62.71 ^ab^	158.68 ^a^	174.92 ^a^	198.08	125.32
CU-FCSL4	3.77 ^b^	44.61 ^b^	86.38 ^b^	111.28 ^b^	251.78	162.22
CU-FCSL6	3.23 ^b^	75.79 ^a^	112.69 ^b^	180.71 ^a^	223.94	137.09
SEM	0.36	4.02	9.97	7.96	18.55	23.16
*p*-value	0.034	0.039	0.002	0.015	0.561	0.732

^a–b^ Means within a row with different superscripts differ significantly (*p* < 0.05). CU-FCSL2: the experimental diets supplemented with 2% corn steep liquor; CU-FCSL4: the experimental diets supplemented with 4% corn steep liquor; CU-FCSL6: the experimental diets supplemented with 6% corn steep liquor; SEM, standard error of the mean.

## Data Availability

The original contributions are contained in this article. Further inquiries can be directed to the first authors.
